# Accuracy of Sedentary Behavior–Triggered Ecological Momentary Assessment for Collecting Contextual Information: Development and Feasibility Study

**DOI:** 10.2196/17852

**Published:** 2020-09-15

**Authors:** Marco Giurgiu, Christina Niermann, Ulrich Ebner-Priemer, Martina Kanning

**Affiliations:** 1 Mental mHealth Lab Institute of Sports and Sports Science Karlsruhe Institute of Technology Karlsruhe Germany; 2 Department of Psychiatry and Psychotherapy Central Institute of Mental Health, Medical Faculty Mannheim Heidelberg University Mannheim Germany; 3 Department of Sport Science University of Konstanz Konstanz Germany

**Keywords:** sedentariness, Ecological Momentary Assessment, accelerometry, mHealth, context

## Abstract

**Background:**

Sedentary behavior has received much attention in the scientific community over the past decade. There is growing evidence that sedentary behavior is negatively associated with physical and mental health. However, an in-depth understanding of the social and environmental context of sedentary behavior is missing. Information about sedentary behavior, such as how everyday sedentary behavior occurs throughout the day (eg, number and length of sedentary bouts), where, when, and with whom it takes place, and what people are doing while being sedentary, is useful to inform the development of interventions aimed at reducing sedentary time. However, examining everyday sedentary behavior requires specific methods.

**Objective:**

The purpose of this paper is (1) to introduce sedentary behavior–triggered Ecological Momentary Assessment (EMA) as a methodological advancement in the field of sedentary behavior research and (2) to examine the accuracy of sedentary behavior–triggered EMA in 3 different studies in healthy adults. Moreover, we compare the accuracy of sedentary behavior–triggered EMA to simulations of random-trigger designs.

**Methods:**

Sedentary behavior–triggered EMA comprises a continuous assessment of sedentary behavior via accelerometers and repeated contextual assessments via electronic diaries (ie, an application on a smartphone). More specifically, the accelerometer analyzes and transfers data regarding body position (a sitting or lying position, or an upright position) via Bluetooth Low Energy (BLE) to a smartphone in real time and triggers the deployment of questionnaires. Each time a participant spends a specified time (eg, 20 minutes) in a sedentary position, the e-diary triggers contextual assessments. To test the accuracy of this method, we calculated a percentage score for all triggered prompts in relation to the total number of bouts that could trigger a prompt.

**Results:**

Based on the accelerometer recordings, 29.3% (5062/17278) of all sedentary bouts were classified as moderate-to-long (20-40 minutes) and long bouts (≥ 41 minutes). On average, the accuracy by participant was 82.77% (3339/4034; SD 21.01%, range 71.00-88.22%) on the study level. Compared to simulations of random prompts (every 120 minutes), the number of triggered prompts was up to 47.9% (n=704) higher through the sedentary behavior–triggered EMA approach. Nearly 40% (799/2001) of all prolonged sedentary bouts (≥ 20 minutes) occurred during work, and in 57% (1140/2001) of all bouts, the participants were not alone.

**Conclusions:**

Sedentary behavior–triggered EMA is an accurate method for collecting contextual information on sedentary behavior in daily life. Given the growing interest in sedentary behavior research, this sophisticated approach offers a real advancement as it can be used to collect social and environmental contextual information or to unravel dynamic associations. Furthermore, it can be modified to develop sedentary behavior–triggered mHealth interventions.

## Introduction

### Growing Awareness of the Risks of Sedentary Behavior

“Sitting is the new smoking” or “Why a sedentary lifestyle is killing you”—these and similar headlines have received a high level of media attention in recent years. There is growing evidence that sedentary behavior is a behavioral risk factor for human health [1]. In particular, researchers identified that too much sitting is a major risk factor for physical and mental health [2,3]. For example, studies indicated that sedentary behavior is associated with cardiometabolic diseases, diabetes mellitus type 2, and mood disorders [4-6]. Since the amount of evidence has been increasing, countries have started to publish public health guidelines for adults to reduce sedentary time [7,8]. However, currently, there are still uncertainties and divergent views on this behavior [9,10], mainly related to inconsistencies in the definition of sedentary behavior and inaccuracies in the measurement of sedentary behaviors. This paper gives a short overview of sedentary behavior definitions and measurement methods, pointing out the currently recommended ones, and introduces sedentary behavior–triggered Ecological Momentary Assessment (EMA) as an innovative measurement approach for measuring contextual information.

### Defining Sedentary Behavior

Several different definitions have evolved over the past decade [11]. From a historical perspective, researchers began by classifying sedentary behavior as physical inactivity. Although sedentary behavior is indeed a form of physical inactivity, the results from physiological studies identified unique mechanisms and characteristics of sedentary behavior and thus suggested that sedentary behavior is an independent behavior, with its own facets and not just the absence of physical activity [12]. Some definitions focused on postural aspects, whereas others focused on energy expenditure without considering postural aspects such as standing versus sitting [11,13], which is questionable since standing may have distinct effects on health outcomes [14-16]. To provide clarity, the Sedentary Behavior Research Network (SBRN) [11] defined sedentary behavior as “any waking behavior characterized by an energy expenditure ≤ 1.5 metabolic equivalents (METs), while in a sitting, reclining, or lying posture.” Unlike other definitions, it comprises both components of sedentary behavior (ie, body posture and movement intensity or energy expenditure). Furthermore, this definition clarifies that standing is not a sedentary state. At this time, the definition of the SBRN is internationally accepted, although there are still some ongoing discussions [13]; for example, the threshold of ≤ 1.5 METs is questionable because the amount of energy expended during sitting does exceed the 1.5-MET threshold in some individuals [10].

### Measuring Sedentary Behavior

Previous studies in the field of sedentary behavior research used self-reported methods such as questionnaires, which have limited validity and are prone to recall biases and social desirability [17,18]. Furthermore, many studies have used television time as a marker of sedentary behavior to examine adverse health effects [19]. However, television time does not reflect all facets of sedentary behavior, and it is confounded by other factors that are relevant for health outcomes such as dietary intake and socioeconomic status [9]. Therefore, based on advancements in device-based measurements, new paradigms suggest using activity monitors [20]. Currently, an increasing number of studies have used device-based measurements of sedentary behavior [17]. However, the choice of monitor placement is highly important for measuring a sitting or lying position versus a standing posture accurately and, therefore, for meeting the definition stated above. Since the inclinometer measures the angle between the gravity direction and the accelerometer's vertical axis, hip-worn accelerometers are limited to distinguishing between sitting and standing. In contrast, thigh-worn accelerometers are recommended as the gold standard [17,21,22]. Some studies have already used thigh-worn accelerometers: For example, the Maastricht study, which focused on the etiology of type 2 diabetes, its common complications, and its emerging comorbidities, assessed sedentary behavior data from approximately 9000 participants via thigh-worn ActivPALs accelerometers [23]. The Prospective Physical Activity, Sitting, and Sleep consortium (ProPASS) provides a detailed overview of existing studies that have used thigh-worn accelerometers [17]. Although the technical possibilities spur constant progress, this research field is still in its infancy. According to the most recent overview of sedentary behavior and health, there is a pressing need to develop further objective field methods for simultaneously assessing both components of the sedentary behavior definition, which is the postural component (sitting or lying) and the movement intensity and energy component [1].

### What is Currently Known About Sedentary Behavior

The latest findings from Stamatakis and colleagues [24] suggest that sedentariness is associated with all-cause and cardiovascular-disease mortality among the least physically active adults. Similar results were found in other epidemiological studies [25,26]. In particular, longer sedentary bouts (ie, a period of uninterrupted sedentary time such as ≥ 30 minutes) may lead to detrimental health effects [27,28].

However, the epidemiological evidence of sedentary behavior' effects on health is incomplete [9]. An important issue is that the majority of studies relied on subjective measures. For instance, a large number of previous studies used self-reported methods such as television time as a marker of sedentary behavior [19,29]. However, Prince and colleagues` [30] meta-analysis revealed that self-reports underestimated sedentary time by 1.74 hours per day in comparison to device-based measures. The evidence for negative health effects relying on device-based measures is sparse as, so far, only very few studies used device-based methods. For example, the EPIC-Norfolk study has shown that sedentary time was associated with cardiovascular disease, cancer, and all-cause mortality [31]. Accordingly, the evidence regarding the adverse effects of sedentary behavior on health should be interpreted in terms of the problems mentioned above, as different definitions and different measurements naturally lead to different results. While it is indisputable that too much sitting is related to risk factors for health, it remains unclear what “too much” is and what the optimal and practical sedentary break patterns are (ie, type, volume, frequency, intensity, and context) that can buffer negative effects. Therefore, further studies with valid device-based measurements are needed [17,20].

Currently, thigh-worn accelerometers are the method of choice for measuring sedentary behavior accurately [17]. However, accelerometers are unable to provide information about the type or the social and environmental context of sedentary bouts. According to the ecological model [32], sedentary behavior is omnipresent in daily life, and it is multifaceted; for example, it can occur during work, leisure-time, household work, or transport. Moreover, in contrast to physical activity, sedentary behavior is invisible, which means that sedentary behavior is merely a procedural subcomponent of purposeful actions such as working, talking, driving, or reading [33]. To understand everyday sedentary behavior and its antecedents and consequences, it is crucial to collect information about social and environmental contexts. Up to now, we have known little about what everyday sedentary behavior looks like, where, when, and with whom it takes place, and what people are doing while being sedentary. Moreover, to develop effective intervention strategies, it is valuable to know more about socioecological mechanisms within different contexts. Thus, with the aim of changing sedentary behavior patterns, subjective information regarding social and environmental contextual information as well as social-cognitive determinants is a valuable extension for the use of activity monitors [10,18,22].

To the best of our knowledge, there is a lack of studies addressing the social and environmental contexts of sedentary behavior. Fortunately, with EMA, there exists an established approach to assess social and environmental context information in daily life [34]. For example, Liao and colleagues [35] used an EMA design to examine where and with whom children's sedentary behavior occurred during non-school time. Their results revealed that children engaged more in leisure-orientated behavior (such as watching television) than productive sedentary behavior (such as homework). Furthermore, most sedentary time occurred at home and in the company with family members. A further EMA study by Romanzini and colleagues [36] examined sedentary behavior contexts among young adults and showed that the context with the highest occurrence of sedentary behavior was the home context—the main activity while being sedentary was “watching TV and movies,” and the main social context was “having alone time.” Such pieces of information may enable researchers to tailor context-specific intervention strategies. However, to assess social and environmental contextual information during sedentary episodes or to know when a meaningful moment to intervene occurs, it is crucial to assess variables or to intervene during predefined sedentary episodes (eg, > 20 minutes) and not during other everyday life episodes, in which the person is physically active, for instance. The umbrella term “just-in-time adaptive interventions” (JITAIs) describes interventions that provide behavioral support that corresponds to a need in real-time when the individual is at risk of engaging in an adverse health behavior such as prolonged sedentariness. In particular, this approach comprises a system that offers “just-in-time” automatic behavioral support without individuals' direct participation [37]. A technical solution for a system that detects, triggers, and collects information about prolonged sedentary behavior is to combine accelerometers and EMA (eg, via applications on smartphones) [38,39]. The sedentary behavior–triggered EMA approach enables researchers to incorporate information from subjective measures (eg, questionnaires) and device-based measures (eg, accelerometers) precisely in those situations where the event (such as prolonged sedentary behavior) occurs.

### Objectives

The purpose of this paper is (1) to introduce sedentary behavior–triggeredd EMA as a methodological advancement in the field of sedentary behavior research and (2) to examine the accuracy of sedentary behavior–triggered EMA in 3 different studies among healthy adults. Moreover, we compared the accuracy of sedentary behavior–triggered EMA to simulations of random-trigger designs.

## Methods

### Sedentary Behavior–Triggered Ecological Momentary Assessment

EMA, sometimes also called the Experience Sampling Method (ESM), is currently a state-of-the-art methodology for examining within-subject associations in behavioral relationships [40,41]. Several advantages (such as the ability to assess in everyday life, in real-time, and repeated measurements with a high sampling frequency) have led to the use of EMA in a wide range of research areas [42]. Currently, technological progress enables researchers to collect data in ways that were inconceivable two or three decades ago. For instance, the combination of EMA and external monitors (eg, accelerometers) provides a wide range of new possibilities, such as triggered EMA, a technical evolution within the EMA methodology. This sampling strategy enables researchers to capture specific behavioral episodes such as prolonged sedentary behavior and to ask participants “just in time” about momentary physical and social contexts or psychological parameters such as mood or stress.

**Figure 1:**
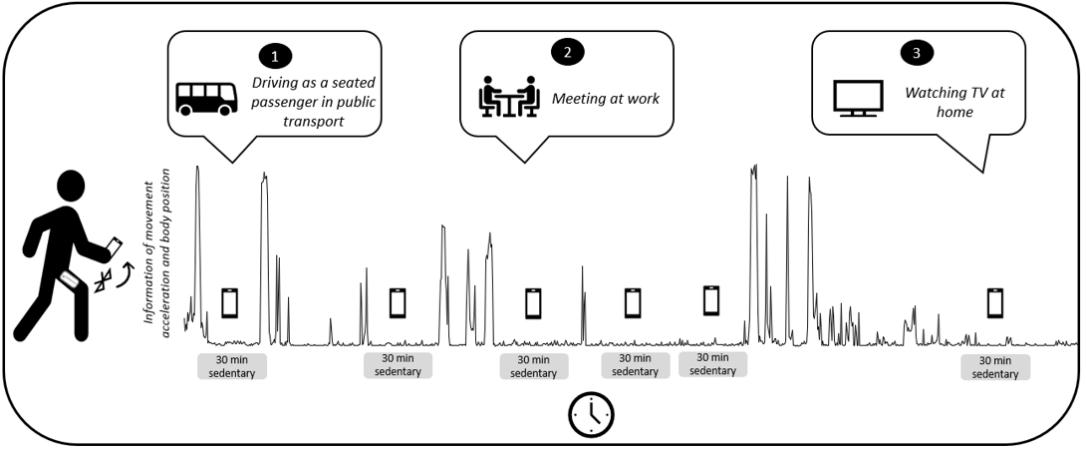
Examples of sedentary behavior–triggered Ecological Momentary Assessment (EMA) in everyday life.

The idea of triggered EMA (or e-diaries) is not entirely new, as Ebner-Priemer and colleagues [39] developed a sophisticated activity-triggered algorithm that focused primarily on physically active episodes in everyday life. Based on similar technical requirements, we developed a sedentary-triggered algorithm for which the following equipment is necessary: a thigh-worn accelerometer (eg, Move 3 Activity Sensor, movisens GmbH), an electronic diary (eg, an application on a smartphone), and a technical interface between the e-diary and accelerometer [eg, Bluetooth Low Energy (BLE)] for feedback in real time. In this study, we used the Move 3 accelerometer (movisens GmbH), which is a single-unit accelerometer that captures movement acceleration and body positions with a measurement range of ±16 g-force (g) at a sampling frequency of 64 Hz. Raw acceleration was stored on an internal memory card. The Move accelerometer has been shown to be a valid device for recording movement behavior [43]. The sedentary behavior–triggered EMA algorithm works as follows: the thigh-worn sensor analyzes data on body position (a sitting or lying position, or an upright position) and transfers the momentary value of the body position in real time to the smartphone. Each time a specific, uninterrupted amount of time spent in a sitting or lying posture is recorded (eg, a 20 or 30 minute period), an e-diary is triggered to begin assessing real-time context information (Figure 1).

### Participant Recruitment and Study Design

We used the sedentary behavior–triggered EMA system in 3 different studies, aiming to examine the accuracy of this approach. Table 1 provides an overview of the different study characteristics.

#### Study 1

We recruited 57 university employees from the Karlsruhe Institute of Technology (KIT) in Germany between May and August 2017. Participants carried a smartphone (Motorola Moto G, Motorola Mobility LLC) and three Move 3 accelerometers for 5 consecutive days. Participants wore accelerometers during the entire measurement period, but not during sleep, swimming, and showering. The thigh-worn monitor and the smartphone were connected via BLE. Sedentary behavior–triggered EMA was used within a mixed sampling scheme. In particular, during the time period from 7:30 am to 9:30 pm, participants received sedentary behavior–triggered prompts (ie, after at least 30 minutes were spent in a sitting or lying position) and randomly triggered prompts at various time points. Since sedentary time is a highly prevalent behavior in daily life, triggered prompts may occur several times per day, which may increase participants' burden. A solution to minimize participants' burden is to implement time-out phases, in which researchers define a time period (eg, of 20, 30, or 40 minutes in duration) when the participants receive no EMA prompts after an answered EMA prompt. During this time-out phase, the study design inhibits EMA prompts, even if the sensor detects an event of uninterrupted sedentary time. In particular, in our first study, EMA prompts occurred no more than every 40 minutes. At each EMA prompt, participants were asked about their social (alone versus not alone) and environmental (home versus work versus leisure activities) contexts (Table 1). Detailed information on the study was described elsewhere [4]. The study was approved by the institutional review board of the Karlsruhe Institute of Technology. All eligible participants received written and oral information regarding the study procedures before written informed consent was obtained. During this procedure, participants were informed of the importance of the smartphone and the accelerometer not losing connection, with a tolerated free-field distance range of approximately 10 meters.

#### Study 2

We recruited 97 individuals from the University of Konstanz in Germany between May and July 2019. Sedentary behavior was assessed for 4 consecutive days (Thursday to Saturday) using Move 3 accelerometers, which were coupled with smartphones (Motorola Moto G, Motorola Mobility LLC) via BLE. During the time period between 6:00 am and 10:00 pm, short questions were asked via the smartphone whenever the person sat for 20 minutes. We implemented a time-out phase of 20 minutes. At each EMA prompt, participants were asked about their social and environmental contexts (Table 1). The participants completed a short paper-pencil questionnaire before the EMA phase that included demographic variables, age, sex, educational level, height, and weight.

#### Study 3

We recruited 72 individuals from the University of Konstanz in Germany between January and March 2019. For 4 consecutive days (Monday to Thursday), participants wore a Move 3 accelerometer on their right thigh from the time they got up in the morning to the time they went to bed in the evening. The accelerometer was connected to a smartphone (Motorola Moto G, Motorola Mobility LLC) via BLE. Prior to the assessment, participants received an extensive briefing on the use of the smartphone and accelerometers and completed a paper-pencil questionnaire that included demographic variables (age, gender, and educational level). During the time period between 6:00 am and 10:00 pm, short questionnaires were asked via the smartphone whenever the person sat for 20 minutes (sedentary trigger). We implemented a time-out phase of 20 minutes. At each EMA prompt, participants were asked about their social and environmental contexts (Table 1).

Data were collected anonymously, and the study fully conformed to the Declaration of Helsinki and the ethics guidelines of the German Psychological Society. Participants received detailed information regarding voluntary participation, the handling of the questionnaires, and the processing of their data, and they gave written informed consent according to the ethics guidelines of the German Psychological Society [44]. According to the guidelines of the ethics committee of the University of Konstanz, the German Research Foundation [45], and the National Science Foundation [46], studies 2 and 3 were exempt from the institutional Ethics Committee review because these 2 surveys were purely observational (noninvasive, noninteractive) and did not induce any type of psychological stress or anxiety, and the participants were not members of a vulnerable group.

**Table 1:** Study characteristics and Ecological Momentary Assessment (EMA) items.

Study characteristics and EMA items				Study 1 (N=57)		Study 2 (N=97)		Study 3 (N=72)
**Characteristics**								
	Duration (days)			5		4		4
	Days of the week			Wednesday-Sunday		Thursday-Sunday		Monday-Thursday
	Valid participants^a^, n (%)			46 (81)		73 (75)		59 (82)
	**Gender of valid participants^a^, n (%)**							
		Male	19 (41)		36 (49)		31 (53)	
		Female	27 (59)		37 (51)		28 (47)	
	Sedentary trigger (minutes)			30		20		20
	Time-out phase (minutes)			Minimum: 40 Maximum: 100		20		20
**EMA items**								
	Environmental context (response options)			*Where are you currently?* (home, work, restaurant, shopping, bus/train, leisure activities, family members, at friends/partners, doctor appointment, exercise, other)		*Where are you currently?* (workplace, canteen, at home, restaurant, bus/train, car, other)		*To which domain would you assign your current sedentary activity?* (work, leisure, home, transport)
	Social context (response options)			*Are you alone at the moment?* (yes, no)		*With whom?* (alone, colleagues, friends, family, strangers,other)		*With whom?* (alone, colleagues, friends, family, strangers, other)

^a^ ≥ 2 days with ≥ 10 hours wear time.

### Study Preparation and Data Preprocessing

**Figure 2:**
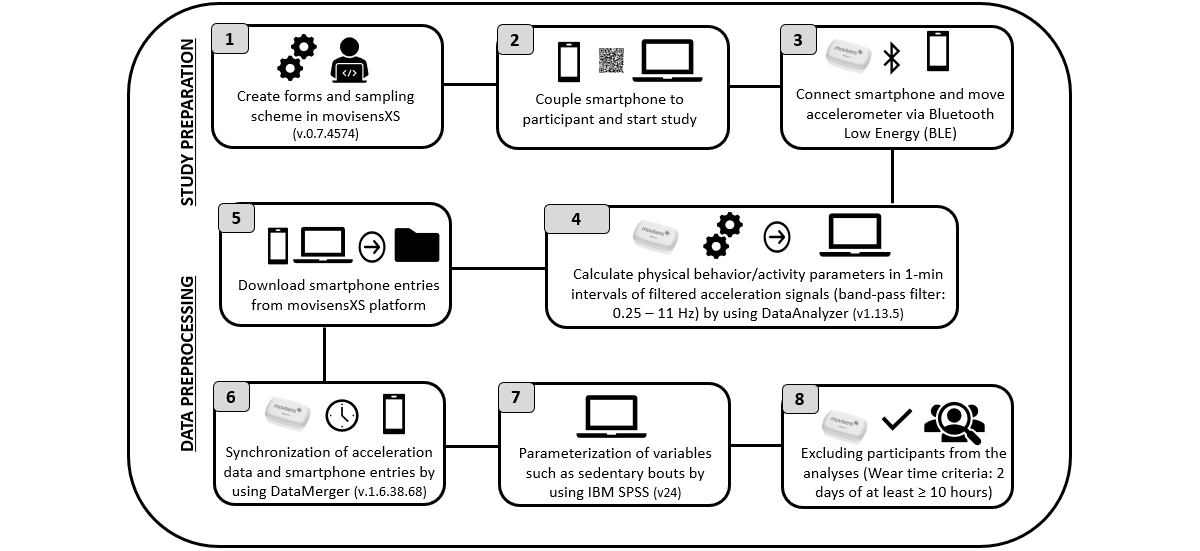
Process of study preparation and data preprocessing.

The same technological system (the Move accelerometer and smartphone with Android operating system) was used in all 3 studies. Thus, from study preparation to data preprocessing, the study procedures (Figure 2) were similar and included the following 8 steps: (1) creation of forms and sampling scheme, (2) coupling of the smartphones to the participants and commencing the study, (3) connection of the smartphones and the accelerometers, (4) processing of the raw acceleration data, (5) downloading the participants' smartphone entries, (6) synchronization of all accelerometer and EMA files into a single data file, (7) parametrization of sedentary-specific variables and calculation of the cumulated sum of the dichotomous variable body position, (8) exclusion of participants who did not fulfill the wear-time criteria (of at least 2 valid days of 10 hours of wear time per day).

First, the sampling scheme and forms (eg, questions about social and environmental context) were created by using the online platform movisensXS (moviesens GmbH). This step included all set-up, such as the selection of the study duration, specification of the trigger option (eg, triggering after 20 minutes or 30 minutes of sitting or lying), and implementation of the time-out triggers. Second, immediately before data collection, the study smartphone was connected to the movisensXS online platform by using the movisensXS app to download the sampling scheme and forms via an individual participant code. Third, the chosen trigger option (eg, triggering after 20 minutes of sitting) was calibrated to the selected body position (the lateral aspect of the right thigh) and connected to the smartphone via BLE by using the movisensXS app. Fourth, after data collection, the recorded raw acceleration data were processed in 1-minute intervals by using the manufacturers' software DataAnalyzer (verson1.13.5, movisens GmbH). During this step, a band-pass filter (0.25-11 Hz) automatically eliminated gravitational components or artifacts (eg, vibrations when cycling on a rough road surface or sensor shocks). This resulted in an Excel spreadsheet with a self-selected choice of parameters such as body position, movement acceleration intensity (MAI), or activity class. Fifth, the smartphone entries from the participants were downloaded from the movisensXS online platform. Sixth, all accelerometer and EMA files from different participants were synchronized and combined into a single data file using DataMerger (version1.8.0, movisens GmbH). Seventh, prior to the analyses, we parametrized sedentary-specific variables such as sedentary bouts while calculating the cumulated sum of the dichotomous variable body position (1= sitting/lying; 0= upright). Eighth, we excluded participants from the data set if they did not fulfill the wear-time criteria of at least 2 valid days of 10 hours of wear time per day [47]. To distinguish wear-time from nonwear time, we used a commercial algorithm and verified its functioning by scanning simultaneously recorded electrocardiogram parameters [48]. Due to the wear-time criteria, we excluded 11 participants from the sample of study 1, 24 participants from the sample of study 2, and 13 participants from the sample of study 3. More details about the technical system used (the accelerometer and online platform) are described elsewhere [4,49].

### Statistical Analysis

To test the accuracy of sedentary behavior–triggered EMA, we calculated an accuracy score, which is the percentage of all triggered prompts in relation to the total number of all possible triggered prompts. In particular, we first calculated sedentary bouts based on the cumulative sum of the dichotomous variable body position (1= sitting/lying; 0= upright) that was recorded by the accelerometer, and categorized them into the following categories: short bouts (≤ 5 minutes), short-to-moderate bouts (5-19 minutes), moderate-to-long bouts (20-40 minutes), and long bouts (≥ 41 minutes). Second, since earlier studies [43,48] have shown that during sitting or lying periods, the physical activity metric MAI [50] did not exceed the 100 milli g-force (milli-g) threshold, we decided to include only moderate-to-long and long bouts in our analyses if the mean MAI of the bouts was < 100 milli-g. Otherwise, sedentary bouts were categorized without considering acceleration intensity; for instance, a 20-minute bout of cycling in a sitting posture would be incorrectly classified as a sedentary bout. Accordingly, we excluded 9.49% (152/1602) of all moderate-to-long and long bouts in study 1, 4.37% (91/2082) in study 2, and 1.94% (32/1649) in study 3. Third, we calculated the accuracy while checking whether an EMA prompt was triggered during accelerometer-recorded moderate-to-long and long sedentary bouts. We also compared the accuracy of sedentary behavior–triggered EMA with that of a purely random trigger design of (1) every 90 minutes and (2) every 120 minutes. Moreover, we conducted additional analyses to test whether demographic factors influenced the accuracy score and explored the social and environmental context of moderate-to-long sedentary bouts.

## Results

### Descriptive Statistics

Table 2 presents the descriptive statistics for each study. Across all studies, we analyzed data from 178 participants, 51.4% (91/178) of which were women and 48.6% (87/178) of which were men, with a mean age of 29.25 (SD 10.51; range 19-66) years and an average BMI of 23.23 (SD 3.1; range 17.1-32.4) kg/m^2^. Across all studies, participants received a total of 10,771 EMA prompts, which was 60.5 (SD 26.5) EMA prompts per participant. On average, participants answered 54.63 (SD 26.32%; range 4.6-100) of the EMA prompts. According to the accelerometer recordings, participants wore the accelerometer 13.96 (SD 1.41; range 10.2-18.6) hours per day. Of that wear time, participants spent an average of 9.5 (SD 1.74; range 5.49-16.57) hours per day in a sitting or lying position. Our data revealed that 29.3% (5061/17,278) of sedentary bouts were classified as moderate-to-long (2488/17,278, 14.4%) and long bouts (2574/17,278, 14.9%); on average, there were 7.67 (SD 1.91; range 1-13) sedentary bouts of ≥ 20 minutes per participant per day.

**Table 2:** Participants' characteristics (N=178).

Variable		Study 1^a^ (n=46), mean (SD; range)	Study 2^a^ (n=73), mean (SD; range)	Study 3^a^ (n =59), mean (SD; range)
Age, in years		34.0 (9.6; 25-62)	28.6 (11.6; 19-66)	26.3 (8.5; 21-60)
**Gender**				
	Female, n(%)	27 (59)	37 (51)	28 (48)
	Male, n(%)	19 (41)	36 (49)	31 (52)
BMI (kg/m^2^)		22.8 (3.3; 17.7-32.1)	23.5 (3.0; 17.1-32.4)	—^b^
Total smartphone prompts^c^		12.31 (1.86; 8-18)	20.72 (7.85; 3-45)	14.83 (5.32; 2-29)
Total triggered prompts^c^		7.3 (2.99; 2-17)	20.72 (7.85; 3-45)	14.83 (5.32; 2-29)
Compliance (%)^d^		79.3 (17.3; 22.2-100)	43.41 (22.5; 4.6-100)	47.49 (23.7; 6.8-93.1)
Wear-time accelerometer (hr/day)^c^		13.6 (1.1; 10.8-16.1)	14.39 (1.6; 10.2-18.6)	13.7 (1.3; 10.7-16.5)
Physical activity of complete measurement period (milli g-force)^c^		86.87 (22.14; 46-148)	81.18 (24.78; 32-141)	79.64 (20.26; 44-155)
Body position: sitting/lying (hr/day)^c^		10.2 (1.6; 7.4-13.7)	9.3 (1.9; 5.7-16.6)	9.16 (1.42; 5.5-12.5)
Total number of short sedentary bouts (≤ 5 min)^c^		11.1 (6.6; 0-29)	10 (6.6; 0-48)	11.9 (6.12; 4-39)
Total number of short-to-moderate bouts (6- ≤ 19 min)^c^		6.3 (2.6; 0-12)	7.1 (2.6; 1-13)	8.6 (3.2; 3-18)
Total number of moderate-to-long bouts (20- ≤ 40 min)^c^		3.5 (1.5; 0-7)	4 (1.8; 0-12)	3.8 (1.2; 2-7)
Total number of long sedentary bouts (≥ 41 min)^c^		4.0 (1.4; 1-7)	4 (1.4; 1-8)	3.6 (1.2; 1-6)

^a^Number of monitoring days per study: Study 1=5 days, Study 2=4 days, Study 3=4 days.

^b^—not available.

^c^Aggregated within the study day per participant.

^d^Percentage of answered Ecological Momentary Assessment prompts across each study sample.

### Accuracy

**Figure 3:**
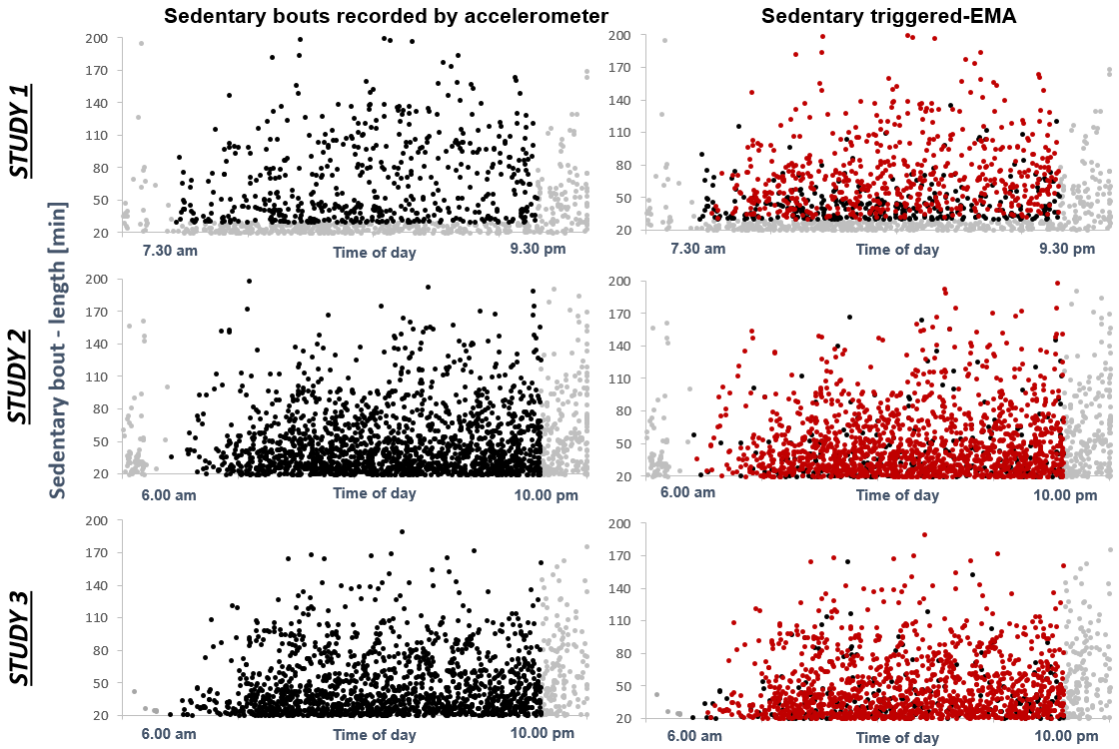
Accuracy of sedentary behavior–triggered Ecological Momentary Assessment (EMA). Left side: the amount of accelerometer-recorded sedentary bouts per study (black dots: sedentary bouts within the study period; grey dots: sedentary bouts outside of the study period). Right side: the amount of triggered EMA diaries (red dots: triggered sedentary bouts; black dots: not-triggered sedentary bouts).

Figure 3 provides a comprehensive overview of the number of accelerometer-recorded sedentary bouts (represented by the black dots on the left side of the figure) as well as a comprehensive overview of the number of bouts that triggered sedentary behavior–triggered EMA (represented by the red dots on the right side of the figure). As a result of a 40-minute time-out trigger in study 1, compared to a 20-minute time-out trigger in studies 2 and 3, there were fewer triggered bouts (red dots) in study 1 than in studies 2 and 3. Moreover, Figure 3 illustrates that the occurrence of sedentary bouts (≥ 20 minutes) is widespread over the day, from morning to evening, in all 3 studies. Overall, 5063 moderate-to-long and long sedentary bouts (≥ 20 minutes) were recorded via accelerometer (Table 3); 11% (559/5057) of these bouts were excluded from the analyses because they occurred prior to or after the study period (ie, 7:30 am - 9:30 pm in study 1, and 6 am - 10 pm in studies 2 and 3). Furthermore, since we implemented a sedentary trigger of ≥ 30 minutes in Study 1, we excluded 32% (464/1450) bouts with a length between 20 and 29 minutes. This resulted in a final number of 4034 sedentary bouts, which could potentially trigger sedentary behavior–triggered EMA. The accuracy calculation revealed that 82.77% (3339/4034) of all possible prompts were triggered. Table 3 summarizes the accuracy on a study level.

Our additional analyses revealed that the sedentary behavior–triggered EMA in the mixed-sampling design of study 1 was 8.97% (n=78) and 20.83% (n=182) higher than that of a simulation of a random-trigger design with prompts every 90 minutes and 120 minutes, respectively. In study 2, the accuracy of the purely sedentary behavior–triggered EMA design was 34.42% (n=587) and 43.46% (n=741) higher than that of a simulation of a random-trigger design with prompts every 90 minutes and 120 minutes, respectively. In study 3, the accuracy of the purely sedentary behavior–triggered EMA design was 34.25% (n=501) and 47.88% (n=699) higher than that of a simulation of a random-trigger design with prompts every 90 minutes and 120 minutes, respectively. These results indicated that the sedentary behavior–triggered EMA system triggered more prompts compared to the simulations of random-trigger designs during moderate-to-long sedentary bouts, and thus, it increases the chance of getting social and environmental context information more often, especially during these kinds of sedentary bouts.

**Table 3:** Accuracy per study.

Measures	Study 1	Study 2	Study 3
Number of all moderate-to-long sedentary bouts (≥ 20 min) recorded via accelerometer	1450	1993	1614
Sedentary bouts prior to 6 am or 7:30 am	19	70	7
Sedentary bouts after 9:30 pm or 10 pm	98	218	147
Sedentary bouts > 20 - < 30 min	464	N/A^a^	N/A
Total number of bouts that could be triggered	869	1705	1460
Triggered sedentary bouts	617	1434	1288
Accuracy of used study design (%)	71.00	84.11	88.22
Accuracy of 90 min. random triggered simulation (%)	62.03	49.69	53.97
Accuracy of 120 min. random triggered simulation (%)	50.17	40.65	40.34

^a^N/A: Not Applicable

**Figure 4:**
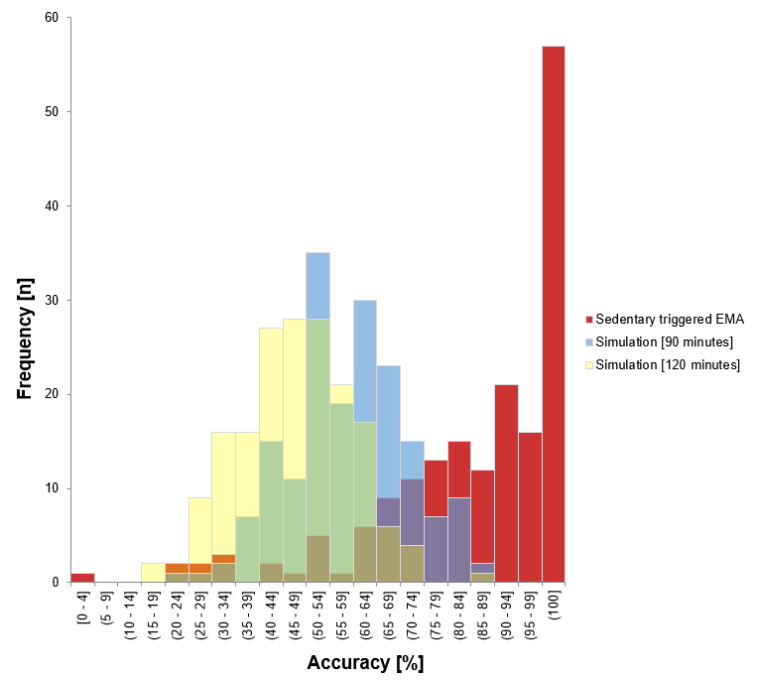
Distribution of subject-level accuracy separated by sedentary behavior–triggered Ecological Momentary Assessment (EMA) design and simulations of random triggered designs of every 90 and 120 minutes.

In addition to the accuracy on the study level, we calculated the accuracy per participant. Figure 4 depicts the distribution of the accuracy on the participant level separated by the sedentary behavior–triggered EMA system and simulations of randomly triggered designs of every 90 and 120 minutes. Data analyses revealed a mean accuracy of 80.90 (SD 20.25%; range 0-100%) for the sedentary behavior–triggered EMA system, a mean accuracy of 54.40 (SD 12.56%; range 18.75-83.87%) for the 90-minute simulation, and a mean accuracy of 43.21 (SD 12.02%; range 10.71-81.8%) for the 120-minute simulation. Additional analyses of a 2-tailed *t* test revealed no significant difference (t_171_= -0.412, *P*=.68) in the accuracy scores for women (80.04, SD 19.59%) and men (81.32, SD 21.37%). Moreover, we found no association between accuracy score and BMI (*r*=0.009; *P*=.92). However, we detected a very small but significant association between accuracy score and age (*r*=-.243; *P*=.001), indicating that the accuracy rate is higher in younger ages.

### Social and Environmental Context

Each time the participants responded to the prompt, they were asked about their current environmental and social context. Across all studies, participants answered 2001 EMA prompts, with an average of 11.57 (SD 7.07) prompts per participant. According to the results about the environmental context, participants reported across all studies that 39.98% (800/2001) of all moderate-to-long and long sedentary bouts occurred during work, 32.93% (659/2001) occurred while at home, 22.44% (449/2001) occurred during leisure activities, and 4.65% (93/2001) occurred during transport. According to the results about the social context, participants reported across all studies that in 56.27% (1126/2001) of all moderate-to-long and long bouts, they were not alone. Specifically, data from studies 2 and 3 revealed that the participants were mostly in the company of friends or family members. Table 4 comprises an overview of the reported results of the environmental and social context by each study on a participant level. The data revealed a high variability between participants. For instance, some participants spent all sedentary bouts during work or while being alone, whereas other participants spent no single sedentary bout during work or while being alone.

In our additional analyses, we found a significant positive correlation (*r*= 0.4; *P*<.001) between age and percentage of being with family members during sedentary bouts. Furthermore, we found significant differences (t_122_=-2.95, *P*=.004) in the percentage of being with family members for women (24.17, SD 28.46%) and men (11.34, SD 24.17%) during sedentary bouts, indicating that the percentage of moderate-to-long and long sedentary bouts in the company of family members increases with age and is higher for women. However, we found no further significant correlation or differences between age, sex, or BMI and percentage of being in environmental domains (ie, work, home, leisure, or transport) or in other social contexts (ie, colleagues, friends, strangers, or others) during moderate-to-long and long sedentary bouts.

**Table 4:** Results of social and environmental context for each study sample.

Participant responses		Study 1, mean (SD; range)		Study 2, mean (SD; range)		Study 3, mean (SD; range)	
Number of answered prompts		11.02 (5.72; 2-26)		11.87 (7.99; 1-30)		11.78 (6.86; 1-25)	
**Environmental context (%)^a^**							
	Work		55.40 (26.64; 0-100)		19.09 (10.43; 0-100)		54.42 (28.85; 0-100)
	Home		24.92 (22.42; 0-100)		51.07 (28.23; 0-100)		14.41 (15.55; 0-67)
	Leisure		18.79 (20.11; 0-92)		22.73 (22.62; 0-100)		25.99 (22.27; 0-86)
	Transport		0.89 (3.03; 0-15)		7.38 (13.78; 0-75)		5.19 (9.82; 0-50)
**Social context (%)^a^**							
	Alone		49.92 (24.90; 0-100)		37.36 (30.61; 0-100)		45.86 (27.73; 0-100)
	With colleagues		N/A^a^		12.18 (19.44; 0-83)		17.94 (24.66; 0-100)
	With friends		N/A		29.59 (29.06; 0-100)		22.21 (22.26; 0-100)
	With family		N/A		23.51 (29.30; 0-100)		11.31 (19.02; 0-100)
	With strangers		N/A		1.96 (7.82; 0-60)		6.85 (13.33; 0-56)
	With others		N/A		1.13 (4.80; 0-33)		2.48 (6.95; 0-33)

^a^Frequency percentage on a participant level for each study.

^b^N/A: not applicable.

## Discussion

### Principal Findings

This paper introduced sedentary behavior–triggered EMA as an innovative methodological advancement in the field of sedentary behavior research and assessed the accuracy of sedentary behavior–triggered EMA in 3 different studies of healthy adults. The results indicated that sedentary behavior–triggered EMA captured 82.77% (3339/4034) of all possible sedentary bouts from the different studies. Compared to simulations of random triggered prompts, our data revealed that the sedentary behavior–triggered EMA system triggered more prompts during moderate-to-long sedentary bouts, and thus it increases the chance for getting social and environmental context information more often, especially during these kinds of sedentary bouts. Overall, the results indicate that sedentary behavior–triggered EMA is an accurate method and allows the capture of “just-in-time” social and environmental context information of sedentary behavior bouts.

### Enhancing Understanding of Daily Sedentary Behavior

Sedentary behavior has received much attention in the scientific community over the past decade. However, in-depth knowledge about this invisible behavior is still missing [9,33]. Since there is a growing number of studies that have found adverse health effects due to sedentary behavior [1], there is now an urgent need to understand more about circumstances surrounding sedentary behavior such as where it occurs, when it occurs, with whom it occurs, and what people are doing while being sedentary. Thus, high-quality assessment methods such as device-based measurements and methods that collect information on domains (eg, work or leisure), types (eg, watching television while sitting), and contexts (eg, being alone or in company) of behavior are recommended by researchers [10,22]. Only a few studies differentiated among context-specific sedentary times, such as Dempsey and colleagues [51], which have shown that higher sitting time was associated with higher levels of individual biomarkers during television viewing and computer use, and lower levels during occupational sitting. In summary, those few studies mainly differentiated between working and nonworking hours [52,53], whereas the social context remained unconsidered. The social context might be relevant as, for example, the social withdrawal hypothesis [54] reported that greater use of the internet (which is mostly related to a sedentary position) was associated with declines in individuals' social interaction and an increase in depression and loneliness. To verify such a hypothesis, sedentary behavior–triggered EMA may be a useful approach for examining both social interaction and mood in real-time during sedentary bouts (eg, internet use).

In general, EMA is an established procedure for the assessment of intrapersonal and social and environmental contextual information, and it has been widely used in previous studies, for example, in the field of physical activity research [55-58]. To the best of our knowledge, there are very few studies that have applied an EMA design in the context of sedentary behavior research [35,36,59-61]. However, these studies used a random time-based, and not a trigger-based, design. Using only a random time-based design may lead to many prompts being issued during situations other than sedentary bouts. At an extreme, not using sedentary behavior–triggered EMA may impede researchers from unraveling existing associations between sedentary bouts and intrapersonal, interpersonal, and environmental variables (such as mood, social interaction, and context) if, by chance, these variables were assessed only during short sedentary bouts or episodes of physical activity but not during prolonged sedentary bouts. Moreover, since our data revealed a high variability of contextual patterns between participants, the sedentary behavior–triggered EMA design increases the chance of capturing situations that are more specific. In other words, not using a sedentary behavior–triggered design increases the risk of an incomplete picture of sedentary behavior since we might miss the more rare events (such as sedentary bouts in public transport). Furthermore, in comparison with random triggered designs, sedentary behavior–triggered EMA minimizes the variance of the bout length, which might be helpful to get more contextual information about specific bout lengths and to examine the health effects of different bout lengths (eg, 10, 20, 30, or 60 minutes).

These are the first studies that used a sedentary behavior–triggered EMA and that assessed social and environmental contextual factors during prolonged sedentary bouts. Sedentary behavior–triggered EMA enables researchers to gather relevant information related to the behavior in real-time. Moreover, sedentary behavior–triggered EMA can also be used to unravel dynamic associations. In particular, future researchers may be interested in discovering dynamic associations between sedentary behavior and possible antecedents and consequences, such as the association between sedentary behavior and time-varying constructs like mood, stress, or working memory. In such a study, it may be reasonable to combine triggered and random prompts to maximize the outcome variance. Furthermore, sedentary behavior–triggered EMA can be modified as a methodological system in a JITAI [37]. For example, each time an individual exceeds a specific threshold of time spent in sedentary behavior (eg, ≥ 30 minutes), mobile apps may deliver behavioral support or encouragement to breakup sedentariness, such as by encouraging an individual to stand up and walk for a few minutes. Finally, a triggered EMA study design minimizes not only retrospective bias but also the burden of participants. In particular, participants would be assessed only in situations in which a behavior of interest occurred (eg, prolonged sedentary behavior).

### Challenges While Using Sedentary Behavior–Triggered EMA

There are also some challenges when using sedentary behavior–triggered EMA. The accuracy depends on both technical stability and user compliance when participating. In particular, technical issues (such as the accelerometer stopping data recording, or the accelerometer and the smartphone losing their BLE connection or not reconnecting with each other) may hinder a functional system. Furthermore, the compliance and reliability of the participant with regards to carrying the smartphone throughout the study period is a critical aspect. For example, if the participant leaves the smartphone at home while he is going to work, the BLE connection would not be available, and the trigger system would not work. This may explain why the accuracy for some participants was very low in our studies. However, short-term disconnections might be a minor issue for future studies since the next generation of accelerometers can store temporary, online, calculated data and transfers that data to the smartphone after a reconnection. Another issue is that if the participant does not wear the accelerometer and puts the sensor on its side (for example, when in a sitting or lying position), this may lead to the incorrect detection of a prolonged sitting bout. A similar problem may occur if the participant did not wear the accelerometer according to the manufacturer's instructions. However, this could be corrected with valid nonwear time algorithms during offline calculations [48]. Moreover, the study design highly influences the accuracy. Using a longer time-out phase, such as in study 1 (40 minutes), led to a reduced accuracy compared to a shorter time-out phase, such as in studies 2 and 3 (20 minutes). In contrast, the compliance of answered EMA prompts was notably higher in study 1 than in studies 2 and 3. Thus, in summary, it is a fine line between collecting as much data as possible and not burdening a participant to the point of decreasing compliance [62]. This is especially true when the outcome of interest is highly prevalent, as is prolonged sedentary behavior [63]. Therefore, depending on the research question, it could be reasonable to incorporate longer time-out phases. Alternatively, to achieve a high level of adherence, researchers may tailor the sampling scheme by reducing the number of items or the number of study days [64]. Finally, sedentary behavior–triggered EMA increases the chance of getting more contextual information (ie, number of prompts, especially during sedentary bouts) but is still dependent on the compliance of the participants. However, it is possible to combine sedentary behavior–triggered EMA with GPS trajectories [56,57] or wearable camera systems [65] to gather more contextual information.

### Conclusions

The results of 3 independent studies revealed that sedentary behavior–triggered EMA is an accurate method for collecting contextual information in daily life. The accuracy of this approach can vary as a function of the study design (eg, time-out triggers), technical stability (eg, connection between the smartphone and accelerometer), and compliance of the participants (eg, following study instructions). Given the growing interest in sedentary behavior research and the lack of knowledge about social and environmental circumstances surrounding sedentary behavior, this sophisticated approach can offer real advancement. Sedentary behavior–triggered EMA can be used to collect social and environmental contextual information or to unravel dynamic associations. Furthermore, it can be modified to develop sedentary behavior–triggered mHealth interventions.

## Abbreviations

BLE: Bluetooth Low Energy
EMA: Ecological Momentary Assessment
g: g-force
JITAI: just-in-time adaptive intervention
MAI: movement acceleration intensity
MET: metabolic equivalent
milli-g: milli g-force
SBRN: Sedentary Behavior Research Network
